# Relationships Between Maternal Selected Metals (Cu, Mg, Zn and Fe), Thyroid Function and Blood Glucose Levels During Pregnancy

**DOI:** 10.1007/s12011-022-03455-5

**Published:** 2022-11-22

**Authors:** WeiYi Zhang, HongPing Liang

**Affiliations:** The Fifth Clinical Medical College of Shanxi Medical University, Taiyuan, 030000 Shanxi China

**Keywords:** Metal elements, Thyroid hormones, Gestational diabetes mellitus, Pregnant woman

## Abstract

The aim of this study were to understand the intake of selected metals (copper (Cu), zinc (Zn), iron (Fe) and magnesium (Mg)) during pregnancy; to detect serum Cu, Mg, Zn and Fe levels in pregnant women; to analyze the relationships among the selected metals, maternal thyroid function and fasting blood glucose (FBG) levels; to investigate the impact of the selected metals and maternal thyroid function on the risk of gestational diabetes mellitus (GDM); and to provide clinical value for the rational intake of the selected metals and iodine during pregnancy to ensure normal fetal development. The population was recruited from pregnant women presenting to the obstetrics outpatient clinic of Shanxi Provincial People's Hospital (February 2021 to April 2022). Selected metal, thyroid hormone (TH (free thyroxine (FT4), free tri-iodothyronine (FT3), and thyroid-stimulating hormone (TSH)) and FBG levels were measured in pregnant women during early, middle and late pregnancy. Covariance analysis was used to analyze the overall trends in selected metal, TH and FBG levels during pregnancy, and binary logistic regression models were used to assess the impacts of the selected metals and thyroid function on the risk of GDM. In addtion, the potential mediation effects of thyroid functions were explored in the mediation analyses. A total of 65 pregnant women were included in this study. Regression models showed that maternal Mg and Cu levels were positively associated with the risk of GDM, conversely, logFT4 was negatively associated with the risk of GDM. Mediation analyses suggested that the associations between the selected metals (Zn, Cu and Mg) and GDM might be mediated by FT3 levels, and that the Cu-GDM and Zn-GDM association could be explained by FT4 levels. Additionally, the Zn-GDM association could also potentially be mediated by the FT3/FT4 ratio. Our findings suggest that Mg, Cu and FT4 levels may act as influencing factors for the development of GDM, and maternal FT3, FT4 and the FT3/FT4 ratio might be the potential mediators of the associations between the selected metals and GDM risk during pregnancy.

## Introduction

Gestational diabetes mellitus (GDM) refers to the onset of any degree of glucose intolerance in women of childbearing age that occurs during pregnancy, resulting in elevated blood glucose [1]. In China, the prevalence of GDM is increasing each year [2]. It has been suggested that the occurrence of GDM may be associated with adverse pregnancy outcomes (preeclampsia, high birth weight, shoulder dystocia, etc.) [3]. The homeostatic imbalance of selected metals (copper (Cu), magnesium (Mg), iron (Fe) and zinc (Zn)) is one of the mechanisms contributing to the pathogenesis of GDM [4, 5]. Epidemiological studies have shown that Cu, Zn, and Fe are associated with insulin sensitivity and resistance and with the pathogenesis of GDM [6]. However, some scholars have found that serum Fe levels are not associated with an increased risk of GDM [7]. In addition, clinical studies have shown that insufficient Mg intake affects glucose metabolism [8]. The inconsistent findings on the relationships between the selected metals and fasting blood glucose (FBG) levels are worth investigating, and the impact of this relationship on GDM risk is unclear; therefore, further validation of the findings and exploration are still necessary.

Normal thyroid function during pregnancy is essential for fetal development (e.g., fetal central nervous system), especially during the first 20 weeks when the fetus does not produce thyroid hormones (THs) [9]. Some studies have identified the important role of THs in glucose metabolism and homeostasis in the body, and thyroid dysfunction is thought to play an important role in the etiology of GDM [10]. However, some scholars have found no significant relationship between THs and GDM [11]. As the available results are controversial, the relationship between THs and GDM needs to be further investigated.

TH homeostasis requires the involvement of multiple proteins and enzymes, which are closely related to the selected metals [12]. Fe deficiency has been reported to be an independent risk factor for isolated hypothyroxinemia in early pregnancy, Zn supplementation is beneficial in maintaining normal free tri-iodothyronine (FT3) levels, and Mg helps to balance TH secretion [13–15]. Previous studies have suggested that Cu, Mg, Zn and Fe levels may be associated with hypo- and hyperthyroidism and the risk of thyroid cancer [16]. Currently, the interrelationships between the selected metals and thyroid function during pregnancy are poorly reported, and the corresponding mechanisms of influence are unclear.

This study was conducted to explore or validate 1) the correlation and overall trend among the selected metals, THs and FBG levels during pregnancy 2) the impact of the selected metals and THs on the risk of GDM and 3) whether THs play a mediation effect on the associations between the selected metals and GDM risk in Chinese pregnant women.

## Materials and Methods

### Study Population

In this study, data from a total of 756 pregnant women who underwent obstetric examinations in the obstetric outpatient department of Shanxi Provincial People's Hospital from February 2021 to April 2022 was collected. The inclusion criteria were as follows: 1) women who were Taiyuan residents; 2) women aged between 18 and 45 years old; 3) women with regular obstetric examinations and delivery in Shanxi Provincial People's Hospital; and 4) women with a singleton pregnancy. On the other hand, the exclusion criteria were as follows: 1) pregnant women with spontaneous abortion; 2) women with a multiple pregnancy; 3) women treated for infertility; 4) parturients who underwent obstetric examinations in our hospital in the first trimester but left the hospital during the second or third trimester; 5) women with prepregnancy diabetes or those with heart or kidney disease, high blood pressure, a family history of diabetes, and a history of cancer; 6) women taking oral contraceptives or other drugs that affect measurements in the first three months of pregnancy (glucocorticoids, dopamine, antiepileptic drugs, etc.); 7) women with human immunodeficiency virus (HIV) or hepatitis B surface antigen positivity; 8) women with a recent blood transfusion; 9) women with recent respiratory, digestive, urinary, cardiovascular, or reproductive system infections; and 10) women with major mental illness, emotional trauma or physical trauma. This study excluded 691 women for the following reasons: 51 were non-Taiyuan residents; 33 with a twin pregnancy; 224 with miscarriage due to various causes; 32 who underwent previous infertility treatment; 234 with a prenatal checkup at our hospital only in the first trimester; and 117 without serum samples in the third trimester. Therefore, only 65 pregnant women participated in the study.

The study population was divided into three groups: T1 (the first trimester group, gestational age < 14 weeks) (*n* = 65), T2 (the second trimester group, gestational age of 14-27 weeks) (*n* = 65) and T3 (the third trimester group, gestational age ≥ 27 weeks) (*n* = 65). A diagnosis of GDM was made at 24-28 weeks of gestation if the fasting blood glucose level, 1-hour oral glucose tolerance test (OGTT) level and 2-hour OGTT level were reached or exceeded 5.1 mmol/L, 10.0 mmol/L, and 8.5 mmol/L (92 mg/dL, 180 mg/dL, and 153 mg/dL), respectively [17]. Seventeen women were diagnosed with GDM during the study period.

All procedures performed in studies involving human participants were in compliance with institutional and/or national research council ethical standards and the guidelines of the Declaration of Helsinki. The research protocol was approved by the Medical Ethics Committee of the Fifth Clinical Medical College of Shanxi Medical University. Informed consents were obtained from all participants.

### Data Collection

This study recorded the detailed information for the pregnant women, including their age, gestational age, height, weight, gestational age at delivery, parity, previous obstetric examinations, medical history, and medication history. The venous blood of the research participants was collected (after fasting for >8 hours and the next morning (07:00-09:00)) later drawn into an anticoagulant tube containing heparin sodium, centrifuged at 3800 rpm for 8 minutes, and lastly, kept in a -80 °C refrigerator. Tested items included the selected metal (Fe, Mg, Cu, Zn), THs (free thyroxine (FT4), free tri-iodothyronine (FT3), and thyroid-stimulating hormone (TSH)), and FBG levels and the determination of biochemical indicators (alanine aminotransferase (ALT), aspartate aminotransferase (AST), unsaturated iron binding capacity (UIBC), etc.).

### Selected Metal (Cu, Mg, Zn, and Fe) and FBG Tests

The serum Zn concentration was measured using the PAPS reagent method with a standard zinc assay kit (GCELL Company, Beijing, China); the serum Cu concentration was measured using the PAESA reagent method with a standard copper assay kit (LEADMAN Company, Beijing, China); the serum Fe concentration was measured using the TPTZ method with a standard iron assay kit (Beckman Coulter Company, California, America); the serum Mg concentration was measured using the dimethylaniline method with a standard magnesium assay kit (Beckman Coulter Company); FBG levels were determined using the hexokinase method with a standard glucose assay kit (Beckman Coulter Company); and serum Fe, Mg, Cu, Zn and FBG levels were determined using a Beckman Coulter AU5800 automated biochemical analyzer. The reference ranges were as follows: Cu: 11-24.4 μmol/L; Mg: 0.6-1.2 mmol/L; Zn: 10.7-17.5 μmol/L; Fe: 11-32 μmol/L; and FBG: 4-6 mmol/L. Samples were pretreated and analyzed according to the manufacturer's instructions. Internal quality control included replicate measurements of the high- and low-concentration quality control samples provided in the manufacturer's kit, and quality control measurements were performed for eachanalytical batch with intra-assay coefficients of variation (CVs) of <10%, ≤3%, <5%, ≤10%, and ≤3% for Cu, Mg, Fe, Zn, and FBG, respectively, and interassay CVs of <15%, ≤5%, <5%, ≤10%, and <5% for Cu, Mg, Fe, Zn, and FBG, respectively.

### Thyroid Function Tests

Original imported kits from Roche Diagnostic Products Ltd. in Germany, as well as a Roche Cobas e601 electrochemiluminescence fully automated tester, were used to determine serum FT3, FT4, and TSH levels, according to the manufacturer's instructions. Among pregnant women in our hospital, the reference ranges for FT3, FT4, and TSH were 3.1-6.8 pmol/L, 12-22 pmol/L, and 0.27-4.20 μIU/mL, respectively. Additionally and according to the manufacturer’s instructions, the intra-assay CVs for serum FT3, FT4, and TSH levels were <6.5%, <5.0%, and <3.0%, respectively; while the interassay CVs were <7.2%, <6.3%, and <7.2%, respectively.

### Statistical Analysis

Statistical analysis was performed in this study using IBM SPSS Statistics for Windows v 25.0 (IBM Corp, Armonk, NY). First, the normal distribution was determined, and one-way analysis of variance (one-way ANOVA) was used to analyze the indicators of normal distribution; a nonparametric test (Kruskal–Wallis test) was used to analyze the indicators of nonnormal distribution. A natural logarithmic transformation [log(X)] was used to normalize the distribution of FT3, FT4 and TSH. Spearman correlation analysis was used to analyze the correlation among the selected metals (Cu, Mg, Zn and Fe), THs (FT3, FT4, TSH and the FT3/FT4 ratio) and FBG levels. An analysis of covariance was used to analyze overall trends in the selected metals, THs and FBG levels during pregnancy. After the exclusion of confounding factors (gestational age, BMI, parity, etc.), binary logistic regression models were used to assess the impacts of Mg, Cu, and logFT4 on GDM risk. *P*<0.05 indicated a significant difference.

Mediation analyses were further performed to evaluate the mediating role of THs in the association of maternal selected metals with GDM risk. The total effect (TE), direct effect (DE), indirect effect (IE), and the proportion of mediation were calculated according to previously described method [18]. The direct effect denoted the effect of seleted metals on GDM risk after controlling for THs, and the indirect effect represented the effect of maternal seleted metals via THs. The ratio of indirect pathways among the total effect was computed to denote the proportion of mediation by THs levels in pregnancy. Covariates controlled in the mediation analyses were in accordance with regression models.

## Results

### Basic Characteristics of the Research Participants

A total of 65 pregnant women were screened in this study, and the basic characteristics are shown in Table 1. Demographic characteristics are expressed as the mean ± standard deviation (SD) for continuous variables and as counts (%) for categorical variables. The mean age of the pregnant women was 29.37 ± 3.23 years. The mean BMI values during pregnancy in T1, T2 and T3 were 22.65 ± 2.97, 25.12 ± 3.12 and 28.27 ± 2.92, respectively. Medical history investigations showed that none of the pregnant women were smoking or drinking alcohol during their pregnancy. A total of 6.2% (*n* = 4), 12.3% (*n* = 8), and 26.2% (*n* = 17) of the participants suffered from gestational anemia, gestational hypertension, and GDM, respectively. The average parity was 1.28. The mean gestational weeks at which the serum samples were collected in the first, second, and third trimesters were 11.31, 23.02, and 30.69 weeks, respectively. The average gestational week at delivery was 37^+6^ weeks.

**Table 1:** Basic characteristics of study population

Characteristics	Mean ± SD or *n* (%)
Maternal age	29.37 ± 3.23
Gestational weeks	
1st	11.31 ± 1.73
2nd	23.02 ± 2.89
3rd	30.69 ± 2.53
BMI	
1st	22.65 ± 2.97
2nd	25.12 ± 3.12
3rd	28.27 ± 2.92
Parity	1.28 ± 0.49
Gestational diabetes mellitus	
Yes	17(26.2)
No	48(73.8)
Anemia	
Yes	4(6.2)
No	61(93.8)
Hypertension	
Yes	8(12.3)
No	57(87.7)
Gestational weeks at delivery	37.80 ± 0.65

Values are mean ± standard deviation, numbers (percentage)

*SD*, standard deviation; *BMI*, body mass index

### Comparison of T1, T2 and T3

In this study, the differences in Cu, Zn, Fe, Mg, TH and FBG levels among T1, T2, and T3 were analyzed (Table 2). Statistical analysis showed that Cu levels showed an increasing trend throughout gestation, while Fe and Zn levels showed a decreasing trend. In the comparison of Cu, Zn and Fe among the three groups, statistical significance was showed between T2 and T3 with T1 (*P* = 0.002, *P* < 0.001, *P* < 0.001). Mg levels were not significantly different among the three groups. The comparison of TH levels found that for T1 and T2, logFT3 and the FT3/FT4 ratio showed an increasing trend. In contrast, logFT4 showed the opposite trend. Except for T2 and T3, logFT3, logFT4, and the FT3/FT4 ratio were significantly different between T2 and T3 with T1 (*P* = 0.003; *P* < 0.001; *P* < 0.001). logTSH was statistically significant between T1 and T2 (*P* = 0.051). In T2 and T3, FBG levels showed an upward trend, and there was a statistically significant difference in FBG levels (*P* = 0.012).

**Table 2:** Comparisons of essential metals, thyroid hormones and FBG among T1, T2 and T3

Variables	T1(*n* = 65)	T2(*n* = 65)	T3(*n* = 65)	*P*-value
Essential metals				
Cu(μmol/L)	26.23 ± 5.32	32.09 ± 4.61	32.52 ± 5.19	< 0.001*
Fe(mmol/L)	19.00 ± 6.63	16.10 ± 6.50	15.25 ± 6.97	0.002*
Mg(μmol/L)	0.80 ± 0.05	0.79 ± 0.07	0.80 ± 0.15	0.906
Zn(μmol/L)	13.27 ± 4.13	10.27 ± 2.33	10.19 ± 2.46	< 0.001*
Thyroid hormones				
logFT3(pmol/L)	0.58 ± 0.10	0.62 ± 0.06	0.62 ± 0.05	0.003*
logFT4(pmol/L)	1.24 ± 0.05	1.14 ± 0.06	1.13 ± 0.07	< 0.001*
logTSH(μIU/mL)	0.16 ± 0.46	0.30 ± 0.24	0.25 ± 0.35	0.051**
FT3/FT4	0.22 ± 0.06	0.31 ± 0.06	0.31 ± 0.07	< 0.001^#^
FBG(mmol/L)	4.68 ± 0.51	4.64 ± 0.70	4.87 ± 0.59	0.014^###^

Values are mean ± standard deviation

*Cu*, copper; *Fe*, iron; *Mg*, magnesium; *Zn*, zinc; *FT3*, free tri-iodothyronine; *FT4*, free thyroxine; *TSH*, thyroid-stimulating hormone; *FBG*, fasting blood glucose

^*^P values for between-group comparison of parametric quantitative data using One Way ANOVA test;

^#^*P* values for between-group comparison of nonparametric quantitative data using Kruskal - Wallis tests

^*^Except for T2 and T3, *P* values for differences among all groups were less than 0.05;

^**^P values for differences between T1 and T2 were less than 0.05;

^###^*P* values for differences between T2 and T3 were less than 0.05

### Correlation Analysis Among the Selected Metals, Thyroid Function and FBG Levels

**Fig. 1:**
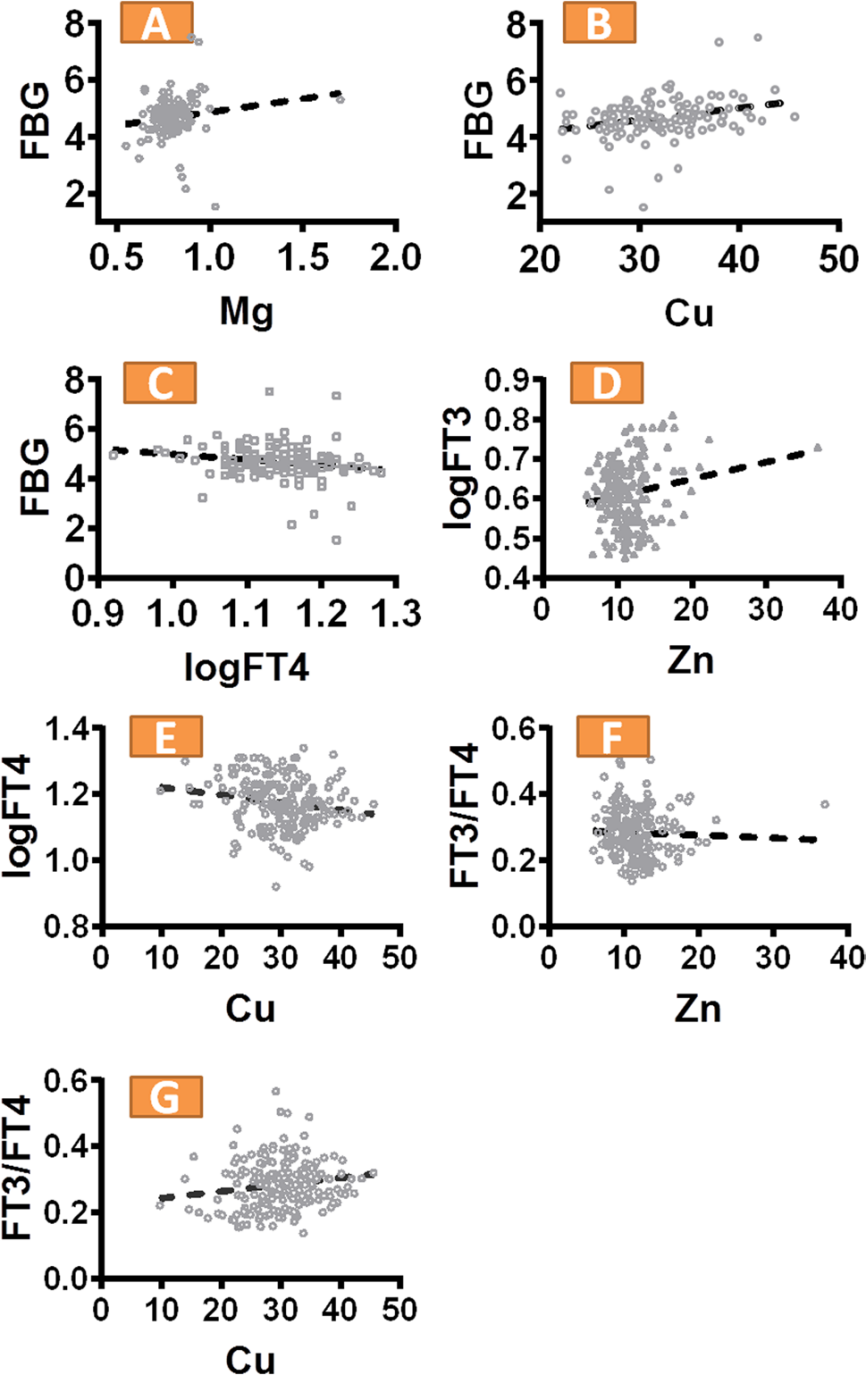
**A**-**G** Correlation between maternal essential metals and log-transformed thyroid function and FT3/FT4. Adjusted for maternal age, gestational weeks, BMI, parity, gestational age at delivery, anemia during pregnancy, hypertensive disorders of pregnancy, gestational diabetes mellitus

Spearman correlation analysis showed that in T2, FBG was positively correlated with Cu, with a correlation coefficient of 0.289 (*P* = 0.020); in T3, FBG was positively correlated with Mg, with a correlation coefficient of 0.247 (*P*=0.048). Elevated Cu and Mg levels were associated with elevated FBG levels, but no correlation was found between Fe and Zn levels with FBG levels. In T2 and T3, logFT4 was negatively correlated with FBG, with correlation coefficients of -0.3 and -0.266, respectively (*P* = 0.015 and *P*=0.032). Elevated logFT4 levels were associated with decreased FBG levels, and no correlation was found between logFT3 and logTSH with FBG levels. Correlation analysis between the selected metals and THs showed that in T1, Cu and Zn were positively correlated with logFT4 and logFT3, respectively, with correlation coefficients of 0.313 (*P* = 0.011) and 0.268 (*P* = 0.031). The FT3/FT4 ratio was used to indicate deiodinase activity. In T1, the FT3/FT4 ratio was positively correlated with the serum Zn level, with a correlation coefficient of 0.369 (*P*=0.002); in T3, the FT3/FT4 ratio was negatively correlated with the serum Cu level, with a correlation coefficient of -0.252 (*P* = 0.043). Elevated Zn levels were associated with increased FT3/FT4 ratios, while decreased serum Cu levels were associated with an increased FT3/FT4 ratio. The analysis showed no correlation between logTSH and the selected metals. The results of the analysis of covariance are shown in Fig. 1. After adjusting for covariates (age, BMI, gestational age at delivery, etc.), analysis of covariance showed that Mg and Cu levels were positively associated with FBG levels during pregnancy (*P*<0.001, Fig. 1A; *P* = 0.015, Fig. 1B); logFT4 levels were negatively associated with FBG levels (*P* = 0.025, Fig. 1C); Zn levels were positively correlated with logFT3 levels (*P* = 0.046, Fig. 1D); Cu levels were negatively correlated with logFT4 levels (*P*=0.032, Fig. 1E); and the FT3/FT4 ratio were positively correlated with Zn levels (*P* = 0.014, Fig. 1F) and negatively correlated with Cu levels (*P* = 0.007, Fig. 1G).

### Regression Models of GDM Risk

The impact of the selected metals on GDM risk in the binary logistic regression analysis is shown in Table 3 while the impact of THs on GDM risk is shown in Table 4. In this study, 65 women were divided into GDM (*n* = 17) and non-GDM (*n* = 48) groups according to whether they had GDM during pregnancy. After adjusting for main confounders, the binary logistic regression analysis showed that serum Cu levels (*P*=0.039, β=1.525), serum Mg levels (*P*=0.018, β=1.594), serum FBG levels (*P*=0.049, β=1.262), age (*P*=0.029, β=1.410), parity (*P*=0.026, β=1.433) and BMI (*P*=0.003, β=0.381) had a significant impact on the risk of GDM and were positively associated. Cu, Mg and FBG levels were positively associated with GDM risk, and the adjusted increase in GDM risk for each unit increase in Cu, Mg and FBG levels was 3.595, 3.923 and 2.532 folds, respectively. Age, BMI and parity were related with GDM risk in a positive manner. Each unit increase in age, BMI and parity were associated with an increase of 3.095, 3.191 and 0.464 folds in GDM risk, respectively. As for the regression model of THs on the GDM risk, logFT4 levels (*P*=0.036, β=-0.631), FBG levels (*P*=0.037, β=1.150), age (*P*=0.045, β=-0.286) and BMI (*P*=0.006, β=-0.015) had a significant impact on GDM risk. The adjusted decrease in GDM risk with one unit increase in logFT4 was 0.788 fold. In addition, one unit increase in FBG level, age and BMI was found to increase GDM risk by 2.158, 1.769 and 0.32 folds, respectively.

**Table 3:** Adjusted binary logistic regression models^a^ for maternal essential metal and related covariates between GDM and non-GDM

	S_b_	Exp(B)	β	*P*
Cu	0.740	4.595	1.525	0.039
Mg	0.675	4.923	1.594	0.018
Zn	0.124	1.003	0.003	0.978
Fe	0.059	1.055	0.054	0.360
FBG	0.641	3.532	1.262	0.049
BMI	0.128	1.464	0.381	0.003
Age	0.645	4.095	1.410	0.029
Parity	0.645	4.191	1.433	0.026

*Cu*, copper; *Fe*, iron; *Mg*, magnesium; *Zn*, zinc; *GDM*, gestational diabetes mellitus; *S*_*b*_, standard deviation; *β*, standardized regression coefficient

^a^Binary logistic regression models were adjusted for maternal age, pregnancy BMI, parity, gestational age at delivery, anemia during pregnancy, hypertensive disorders of pregnancy, gestational weeks

**Table 4:** Adjusted binary logistic regression models^a^ for maternal thyroid hormones and related covariates between GDM and non-GDM

	S_b_	Exp(B)	β	*P*
logFT4	0.739	0.212	–1.549	0.036
FBG	0.552	3.158	1.150	0.037
Age	0.509	2.769	1.018	0.045
BMI	0.101	1.320	0.278	0.006

*FT4*, free thyroxine; *FBG*, fasting blood glucose; *BMI*, body mass index; *S*_*b*_, standard deviation; *β*, standardized regression coefficient

^a^Binary logistic regression models were adjusted for maternal age, pregnancy BMI, parity, gestational age at delivery, anemia during pregnancy, hypertensive disorders of pregnancy, gestational weeks

### Mediation Effects of Maternal THs

In the present mediation analyses, maternal FT3, FT4, and the FT3/FT4 ratio were selected as potential mediators for the association between the selected metals and GDM risk based on the assumption of mediation analysis. Maternal TSH levels did not meet the criteria for mediation analysis and thus not included. As shown in Table 5, the association between Zn levels and GDM risk was significantly mediated by decresed FT3 levels (IE, -0.0123; 95 % CI: -0.0357,-0.0008). Additionally, values of maternal FT3 were found to mediate the associations of Cu and Mg with GDM risk; values of maternal FT4 were found to mediate the associations of Cu and Zn with GDM risk. Values of the FT3/FT4 ratio could mediate (IE, -0.0107; 95 % CI: -0.0308,-0.0001) the associations between Zn and GDM risk.

**Table 5:** Mediated effects by thyroid hormones on associations of selected metal concentrations with GDM^a^

Metals and GDM	Mediators	Direct effects (95%CI)	Indirect effects (95%CI)	Total effects (95%CI)	Proportion of mediation (%)
Mg-GDM	FT3	0.0634 (–0.1276,0.2544)	–0.0506 (–0.1506,–0.0088)**	0.0128 (–0.1782,0.2039)	
Mg-GDM	FT4	0.9449 (–0.1169,2.0066)	0.1345 (–0.0155,0.4381)	1.0793 (0.0289,2.1298)	12.46%
Mg-GDM	FT3/FT4	1.2393 (0.2173,2.2613)	–0.1099 (–0.4814,0.0195)	1.1294 (0.1122,2.1466)	
Cu-GDM	FT3	0.0241 (–0.0029,0.0511)	–0.0085 (–0.0250,–0.0009)**	0.0156 (–0.0113,0.0425)	
Cu-GDM	FT4	–0.3574 (–0.6099,–0.1049)	0.0606 (0.0041,0.1613)**	–0.2969 (–0.5506, –0.0431)	
Cu-GDM	FT3/FT4	0.0028 (–0.0187,0.0244)	0.003 (–0.0001,0.0110)	0.0059 (–0.0155,0.0272)	50.85%
Zn-GDM	FT3	0.004 (–0.0283,0.0362)	–0.0123 (–0.0357,–0.0008 )*	–0.0083 (–0.0395,0.0228)	
Zn-GDM	FT4	0.0068 (–0.0260,0.0395)	–0.0081 (–0.0202, –0.0011)**	–0.0013 (–0.0334,0.0308)	
Zn-GDM	FT3/FT4	0.0024 (–0.0308,0.0355)	–0.0107 (–0.0308,–0.0001)**	–0.0083 (–0.0405,0.0239)	

*Cu*, copper; *Mg*, magnesium; *Zn*, zinc; *FT3*, free tri-iodothyronine; *FT4*, free thyroxine; *GDM*, gestational diabetes mellitus

^a^Maternal thyroid hormones were log-transformed in the models. Mediation models were adjusted for maternal age, pregnancy BMI, parity, gestational age at delivery, anemia during pregnancy, hypertensive disorders of pregnancy, gestational weeks

^*^P < 0.05

^**^P < 0.01

## Discussion

As a follow-up study of 65 pregnant women, this study provides reliable results to investigate the correlation among the selected metal, TH and FBG levels and the impact of the selected metals and THs on the risk of GDM during pregnancy.

This study showed that Mg levels in the third trimester were associated with FBG levels; elevated Mg levels were positively associated with an increased risk of GDM. However, through a systematic review and network meta-analysis, Jin et al. found that an increased intake of Mg reduced serum insulin levels [19]. However, there are also studies that found no significant association between Mg levels and GDM risk [20, 21]. Some scholars found that Mg can protect and repair islet β cells. Mg deficiency can cause changes in pancreatic cell structure, reduce β-cell granules, and lead to insufficient insulin synthesis and secretion. Therefore, changes in Mg levels can affect islet responses [22]. Mg2+ is an important cation and coenzyme involved in enzymatic reactions [23]. Studies have shown that Mg2+ is considered the second messenger of insulin and plays an important role in glucose metabolism stability and insulin sensitivity: i) Mg deficiency reduces insulin receptor activity, leading to insulin resistance and ii) hypomagnesemia inhibits glucose utilization in basal and insulin-stimulated states [24]. The inconsistent results may be due to the small sample size of women with GDM and differences among participants. Although serum Mg levels depend on dietary intake, urinary Mg excretion and intracellular Mg concentrations are more reflective of Mg status than serum Mg levels due to differences in the gastrointestinal and renal function of participants [25]. Therefore, serum Mg levels are less sensitive for assessing Mg status, and the results of this study cannot definitively explain the relationship between Mg levels and GDM risk.

The present study showed that increased Cu levels in the second trimester were positively correlated with FBG levels and that elevated Cu levels were associated with increased GDM risk. In fact, other scholars have similarly reported that the Cu concentration of pregnant women with GDM is higher than that of non-GDM women and that elevated Cu levels were correlated with elevated FBG levels [26]. Possible mechanisms of Cu in the pathogenesis of GDM are as follows: i) The increased requirement for SOD and cytochrome c oxidase, which are maternal Cu-dependent enzymes, may be the reason for the rise in Cu absorption during pregnancy. ii) Dietary habits may change during pregnancy. Consuming more foods high in Cu (e.g., animal products) is more common among pregnant women. iii) Circulating estrogen levels in healthy adults may affect a number of indicators often used to determine the nutritional status of Cu (e.g., serum Cu and ceruloplasmin), and Arredondo et al. have confirmed these findings [27–29]. Therefore, increased circulating Cu concentrations were found in gestational women compared to non-pregnant females, which may be related to hormonal changes during pregnancy. Akhlaghi et al. found no association between Cu and GDM [7]. The reasons for the inconsistency with the results of this study may be related to different dietary habits and ethnic differences in different countries. Cu is mainly derived from the daily diet based on animal protein (e.g., grains, beans), which is consistent with the dietary habits of Asians, and sufficient Cu intake can be obtained in this manner. However, Europeans, who mainly eat meat and dairy products, have little change in Cu intake during pregnancy [26]. This may lead to differences in Cu levels between Asian and European populations.

This study found no correlations between Fe or Zn levels and FBG levels. In related studies, Wilson and Choi et al. found that there was no significant correlation between GDM and serum Zn levels [28, 30]. However, studies have shown an association between Zn or Fe levels and the risk of GDM [21]. Fe is one of the most potent oxidants to induce insulin resistance and interfere with insulin release from pancreatic β cells and is involved in the pathogenesis of GDM; Zn promotes the development of GDM by interfering with insulin metabolism and glucose homeostasis [31, 32]. Decreases in Zn levels are associated with disproportionate increases in plasma volume and transfer from a mother to her fetus [33]. Therefore, the failure to measure plasma volume in this studymay be one of the reasons for the inconsistent results.

This study found that in the second and third trimesters, decreased FT4 levels were associated with increased FBG levels, FT4 levels were negatively associated with GDM risk, and FT3 and TSH levels were not associated with GDM risk. Similar to the results of this study, some scholars found that the FT4 levels in pregnant women with GDM were significantly lower than those in healthy pregnant women [10]. A prospective study by Bell et al. found that TSH and FT3 levels were not associated with the risk of GDM [34]. In a Finnish birth cohort study, Männistö et al. found that overt hypothyroidism was associated with the risk of GDM [35]. THs have been reported to regulate hepatic gluconeogenesis, intestinal glucose absorption, and glucose uptake in peripheral tissues. In addition, THs regulate the messenger RNA and protein expression of glucose transporters, accelerate glycogenolytic pathways, and then alter circulating insulin levels [36]. Some studies have speculated that the inverse relationship between FT4 levels and GDM risk may be due to increased conversion of FT4 to FT3 or increased deiodinase activity [37]. The FT3/FT4 ratio is a common method for judging the transformation of FT4 to FT3. Some researchers have found that the FT3/FT4 ratio is an independent risk factor for GDM and is closely related to GDM [10]. However, this study did not find an association between the FT3/FT4 ratio and GDM risk. Some scholars have also found that women with primary hypothyroidism do not have an associated risk of GDM [38]. The inconsistent results are due to differences in the underlying iodine deficiency of the study population, as iodine is required for the synthesis of THs, as well as differences in ethnic, genetic, and environmental factors, all of which make comparisons between study results difficult. At the same time, the relatively small number of GDM patients also limits the statistical power to analyze significant associations [10, 34].

In the current study, maternal FT3, FT4 levels and the FT3/FT4 ratio were included in the analyses of mediation, and it was discovered that these factors mediated the correlations of maternal Zn, Cu, and Fe levels with the risk of developing GDM. Similarly to our findings, a recent birth cohort study demonstrated that maternal FT3, FT4, and the FT3/FT4 ratio were the mediators in the pathway associating metals with adverse pregnancy outcome. The author assumed that the adverse effects of metals, such as thyroid inflammation and deiodinase activity inhibition, may be attributed to the possible mediation effects of the FT3/FT4 ratio and FT3 [39]. Previous studies have reported a relationship between Zn levels and TH levels. In a study of 531 pregnant women in northern Sweden, Gustin et al. found that low Zn levels were associated with FT3 levels in an adjusted regression model [40]. This study further verified that there is a relationship between Zn and TH levels: An appropriate amount of Zn supplementation during pregnancy can increase TH levels to ensure the normal development of the fetus. Zn is involved in the biosynthesis of thyroid-stimulating hormone-releasing hormone (TRH), and Zn deficiency reduces TH levels [14]. However, some scholars have found that serum Zn levels during pregnancy are positively correlated with FT4 levels, but this study did not find the similar results [41]. The inconsistencies in the results may be due to differences in geographic location, ethnicity, etc. Geographical differences (content of soil elements) lead to differences in the contents of metal elements in organisms, which in turn lead to long-term differences in human eating habits [42]. These differences in eating habits may lead to different results in different populations.

Pop et al. studied the relationship between plasma minerals (Cu, Zn, etc.) and THs in 2041 pregnant women in the Netherlands in their first trimester, and after adjusting for relevant variables, they found that Cu levels were positively correlated with FT4 levels [41]. Jain et al. evaluated the impacts of serum Zn and Cu levels on thyroid function in pregnant American women and obtained similar results [43]. This study further verified the relationship between metal elements and thyroid function; that is, Cu levels were positively correlated with FT4 levels. Some studies have found that serum Cu levels tend to increase during pregnancy, mainly due to maternal estrogen-stimulated increases in ceruloplasmin levels and decreased bile Cu excretion, resulting in a significant increase in Cu concentrations [44, 45].

Clinically, the FT3/FT4 ratio is used to express the conversion rate of FT4 to FT3, which is used to evaluate deiodinase activity [46]. Zn is essential for T3 and nuclear receptors and is mainly involved in the production of TRH; in the transformation of T4 and T3, Zn acts as a cofactor and participates in the monodeiodination of T4 to activate T3, which is catalyzed by type I and type II deiodinases (D1 and D2) [14]. The present study found that an increase in the Zn concentration was positively correlated with an increase in the FT3/FT4 ratio; Cu levels were negatively correlated with the FT3/FT4 ratio, which is consistent with previous findings [39]. Cu stimulates TSH secretion by participating in the synthesis of phospholipids, and Cu deficiency reduces TH levels [47]. Clinical experiments have shown that reducing serum deiodinase activity affects the conversion of peripheral T4 to T3, resulting in Cu deficiency, which is contrary to our findings, possibly due to geographical differences in the study population and differences in thyroxine supplementation during pregnancy. Approximately 2-3% of pregnant women are hypothyroid during pregnancy, so most pregnant women take thyroxine supplements during pregnancy [48]. In this study, we did not investigate the pregnant women’s supplement intake.

Wu et al. found that Fe and Mg levels in pregnant women were negatively correlated with TSH levels and positively correlated with FT3 levels, FT4 levels, and the FT3/FT4 ratio, and Fe deficiency reduced serum FT3 and FT4 concentrations and TPO activity [39, 49]. On the one hand, Fe promotes the effective utilization of iodine; on the other hand, Fe is one of the essential elements for the formation of heme, which is involved in the synthesis of thyroid peroxidase. Once Fe is deficient, TH metabolism becomes disordered [14]. Mg participates in the deiodination process as an enzymatic cofactor to aid in the reduction process involving the electron transport chain [50]. In this study, we found no correlations between maternal Fe or Mg levels and TH levels. The differences in the results may be due to differences in base metal concentrations and differences in study design. In this study, the concentrations of Mg (0.80 mmol/L) and Fe (19.25 μmol/L) were lower than those reported in other reports (1.34 mmol/L for Mg and 7.60 mmol/L for Fe) [39]. Furthermore, we did not administer a questionnaire survey on whether pregnant women took supplements containing the selected metals during pregnancy, and inconsistent results are possible.

The strengths of this study were as follows: 1) This was a follow-up evaluation exploring overall trends in the selected metals and thyroid function during pregnancy and 2) The selected metals (Cu, Fe, Zn, etc.) were included in the study design along with TH levels and GDM risk. At the same time, the study has the following limitations: 1) The sample size was small. Studies with larger sample sizes need to be conducted to elucidate the clinical value of the selected metal concentrations; the small number of pregnant women with GDM limits the statistical power to analyze significant associations. 2) Studies have found that serum mineral levels (e.g., Zn and Cu) are influenced by the systemic inflammatory response, while normal pregnancy is an inflammatory state (as evidenced by cytokines) [51, 52]. Therefore, the selected metals do not truly reflect the pregnancy state. 3) All the participants of this study were

located in western China, and the generalizability of this study to other populations remains to be determined. 4) Even though key confounders were controlled, other confounders (dietary assessment during pregnancy, physiological changes, supplement intake, etc.) were not investigated, and additional factors need to be further considered. 5) Related indicators were determined; however, this study only measured FBG levels, not glycosylated hemoglobin and TPO-antibodies. Due to the small sample size of the current study, the sample size can be expanded in the future to further explore the potential mediating effect of THs between selected metals and GDM risk; in addition, previous research results support the impact of metal elements other than iodine on TH levels and GDM risk, and more micronutrients could be included in research in the future.

In conclusion, in this study, maternal Cu, Mg, and FT4 levels were significantly correlated with FBG levels; Cu and Zn levels were significantly correlated with TH levels and the FT3/FT4 ratio. Maternal Mg and Cu levels may serve as risk factors for GDM onset, and FT4 levels may serve as protective factors for GDM onset. In addition, maternal FT3, FT4 and the FT3/FT4 ratio might be the potential mediators associated with the selected metals-GDM risk during pregnancy.

## Data Availability

The datasets generated during and/or analysed during the current study are not publicly available due to [individual privacy could be compromised] but are available from the corresponding author on reasonable request.
